# A GRASP-Based Approach for Planning UAV-Assisted Search and Rescue Missions

**DOI:** 10.3390/s22010275

**Published:** 2021-12-30

**Authors:** Casper Bak Pedersen, Kasper Gaj Nielsen, Kasper Rosenkrands, Alex Elkjær Vasegaard, Peter Nielsen, Mohamed El Yafrani

**Affiliations:** Department of Materials and Production, Aalborg University, 9220 Aalborg, Denmark; cbpe17@student.aau.dk (C.B.P.); kgni20@student.aau.dk (K.G.N.); krosen17@student.aau.dk (K.R.); aev@mp.aau.dk (A.E.V.); peter@mp.aau.dk (P.N.)

**Keywords:** Search and Rescue, Unmanned Aerial Vehicles, Greedy Randomised Adaptive Search Procedure

## Abstract

Search and Rescue (SAR) missions aim to search and provide first aid to persons in distress or danger. Due to the urgency of these situations, it is important to possess a system able to take fast action and effectively and efficiently utilise the available resources to conduct the mission. In addition, the potential complexity of the search such as the ruggedness of terrain or large size of the search region should be considered. Such issues can be tackled by using Unmanned Aerial Vehicles (UAVs) equipped with optical sensors. This can ensure the efficiency in terms of speed, coverage and flexibility required to conduct this type of time-sensitive missions. This paper centres on designing a fast solution approach for planning UAV-assisted SAR missions. The challenge is to cover an area where targets (people in distress after a hurricane or earthquake, lost vessels in sea, missing persons in mountainous area, etc.) can be potentially found with a variable likelihood. The search area is modelled using a scoring map to support the choice of the search sub-areas, where the scores represent the likelihood of finding a target. The goal of this paper is to propose a heuristic approach to automate the search process using scarce heterogeneous resources in the most efficient manner.

## 1. Motivation

Due to the increased computation and automation capabilities of systems during the last decades, research and industry have shown a high interest in UAV-assisted SAR problems. SAR missions can be triggered due to various events which could be due to natural, technical, political or unknown causes. Examples include hurricanes, earthquakes, tsunamis, wildfires, lost vessels at sea and warfare. SAR problems are divided into two procedures: search (locating targets) and rescue (providing aid). The urgency and contingency of these situations makes it critical to be able to act fast and effectively and efficiently utilise the available resources.

In the context of this study, the search is managed using UAVs, which typically require less infrastructure to deploy compared to other types of vehicles and have a higher degree of manoeuvrability [1]—which is a necessity in most SAR problems—but have a limited battery and payload capacity [2,3]. This makes them suitable for search and potentially for providing first aid (this highly depends on the drone model, mainly its payload capacity [4,5]), but less useful for the rescue part. Note that this is dependent on the specific aircraft in question, as there are many different types of both rotary-wing, fixed-wing, and hybrids models, as well as UAVs specific for different altitudes, e.g., the high-altitude pseudo satellites.

**Figure 1:**
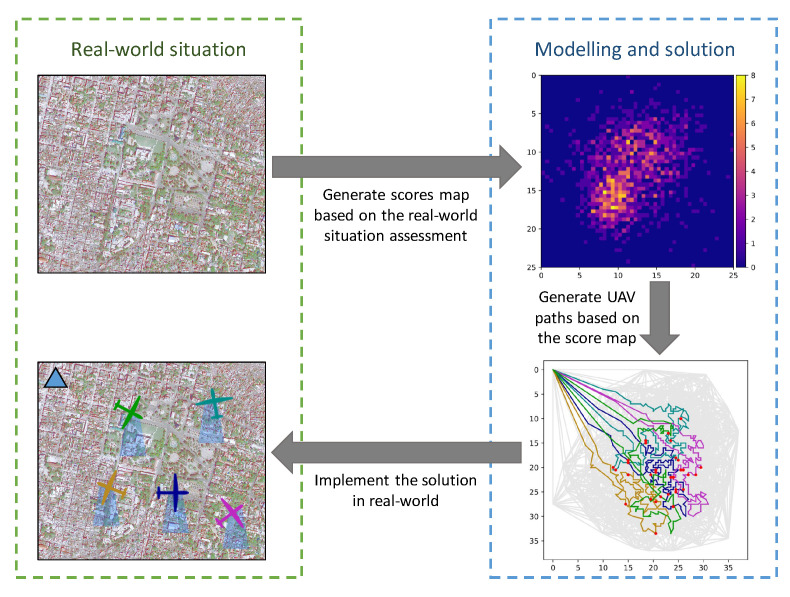
An overview of the process of disaster response and SAR mission planning.

Information about the location of targets is essential for the success of SAR missions, but for these types of missions, this information is often represented as a discrete probability map segmenting the search space into a grid with the value of each cell corresponding to an assigned probability of containing the target. However, probabilities have interpretive properties, which complicates the analysis. Often, the likelihood of finding a target is quantified using scores. The search part of the problem is represented as a complex network, where the goal is to find the feasible routes that maximise the cumulative score for the fleet of UAVs. Feasibility is defined through the usage of battery life, but other factors could also influence the search duration and worthiness of searching a given region. A macroscopic view of the steps involved in planning SAR mission is illustrated in Figure 1. The figure shows the main steps taken in the transition from a real-world disaster situation to a model, on whose basis a solution can be generated and implemented as a response to the triggering event. This study can be scoped within the “modelling and solution” blocs.

In this research, randomly generated maps containing both target locations and scores that represent the likelihood of finding a target in that area are utilised. The maps are assumed to be static (time independent) and will therefore be relevant for missions in low-dynamics environments such as post-disaster SAR. Nevertheless, the solution framework can be adapted or extended to dynamic maps. This is possible due to the employment of heuristics which can return a solution within a specified tight time budget and by considering a sequence of problems to solve. Furthermore, the dependencies between the problems can be managed as the subsequent problems can be constrained based on the outcome of the prior problems.

It is worth noting that most studies focus on map partitioning (e.g., grid maps, segmentation, etc.) and search patterns (e.g., parallel, spiral, etc.) While this has the advantage of simplifying the problem and of potentially reducing conflicts between UAVs, it also limits the fleet’s capability to conduct an efficient search, especially in the case of a heterogeneous fleet, i.e., UAVs have different capabilities. To tackle this, the problem is modelled using a highly connected network. (A highly connected network is defined as a network where the number of arcs on each node is close to the total number of nodes in the network.) of nodes representing the areas of interest, and the goal is to solve the underlying routing problem to visit areas with the highest probability of finding the targets. In addition, San Juan et al. [6] present various approaches for UAV path planning in SAR situation. However, the limited range of a given UAV is not taken into account. In this study, the more realistic scenario of limited range is tackled directly in the mathematical formulation. Furthermore, to the best of our knowledge, SAR missions have not been framed as TOPs, but solutions of these using GRASP can be seen in [7], though the proposed implementation using hill climbing differs significantly.

This research builds upon the previous work in Rosenkrands et al. [8]. Compared to the previous contributions, focus is on the exploration and application of the algorithms, primarily the metaheuristic solution approach. Furthermore, a broader experimental section containing numerical results for a range of different problem instances is presented. Furthermore, it is assumed that UAVs are equipped with optical sensors that are recording and transmitting images in near real-time to a base station where the mission is monitored. SAR missions typically rely on a human agent to monitor the entire mission (Human-In-The-Loop), even when the system has automatic object recognition capability. UAVs also have a limited range which can limit their ability to explore more of the search area. The aim of this paper is to design an autonomous path planning method in a UAV-assisted SAR setting. In real-time, the method outputs routes for the entire fleet of UAVs, and the inputs are a map containing regions of interest, a desired time horizon, and a selection of the fleet of heterogeneous UAVs. Thus, the problem is formulated as a Team Orienteering Problem (TOP) [9] where the search area is represented as a graph uncompressed using the Delaunay triangulation [10]. A greedy algorithm and a hill climbing heuristic are presented. Then, these two methods are used as building blocks to design a Greedy Randomised Adaptive Search Procedure (GRASP) as the main solution approach to the SAR model.

This paper is organised as follows. Section 2 provides background information and a literature review on mission planning and control. Section 3 presents the mathematical formulation which shows the problem and restrictions that the solution methods must consider. Section 4 introduces the solution methods for the aforementioned problem. The experiments, results and analysis are discussed in Section 5, including the impact of the algorithm’s parameters. Finally, Section 6 concludes the paper and revisits some future research and improvement directions.

## 2. Literature Review

This section covers relevant literature on the different map formulation methods, a discussion on the cell value assignment through scoring or probability maps and whether the information is crisp or fuzzy, how the multi-objective framework is considered, whether static or dynamic maps are considered, before lastly different formulations are explored.

The area to be searched can be structured in different ways. A frequently applied approach was presented by Berger et al. [11], where a two-dimensional grid of *N* cells represents the physical search area. However, only maps of 10×10 cells where considered to allow for a comparison between myopic heuristics and exact solutions, where the latter usually is not able to manage larger problem instances. Nielsen et al. [12] proposed a decomposition method given an area of interest represented as a polygon into convex sub-polygons to simplify coverage problems using a back-and-forth search pattern. To emphasise the implementation of heuristics, other papers work with elementary graphs, e.g., complete graphs where the added connectivity means that exact solution methods are unable to solve any problems with real-world complexity.

When given a structure for the map, a very important consideration is the assignment of relative value to each cell, which indicates the attractiveness to visit the specific cell compared to other cells. In the works of San Juan et al. [6], the map of terrain and emergency conditions and the risk/occupancy map are combined and introduced to the system. This yields a nuanced path but also requires significantly more computational power and information retrieval to conduct; similar understanding of the decision process can be difficult, and in SAR missions, ambiguity is not taken lightly, so often this approach is more suitable for fixed SAR systems.

As a continuation of this, how to integrate the different preferences is considered greatly in multi-objective (MO) formulations of the UAV problem [13]. Whether preferences are introduce a priori or a posteriori through scoring or by estimating a Pareto front, respectively [14]. Estimating Pareto fronts is very time consuming, so to accommodate this, Atencia et al. [15] developed a MO evolutionary algorithm integrating a weight generator to narrow the solution space. In SAR settings, time is of the essence, so often a scoring method is applied.

Most of the literature considers static maps, but for specific SAR scenarios, it could be highly valuable to consider dynamic changes, e.g., if a high-speed target, a slow search procedure, a large search area or a rapidly changing environment is considered.

The mathematical formulation by Berger et al. [11] accounts for the possibility that a target is not necessarily spotted when the UAV visits the specific cell, thereby, incorporating the randomness to allow multiple visits to the same cell. Another approach by Evers et al. [16] is by evaluating the arc weight through fuzzy estimates and utilising stochastic programming or robust optimisation to deal with the uncertainty. A reason for this could be poor visibility in the environment with high fog or dense foresting or, in the case of thermal cameras, if the target has the same temperature as the surroundings.

The Orienteering Problem (OP) presented by Golden et al. [17] considers only one agent, but it was later generalised to include multiple agents [9], since being referred to as the TOP. The case with multiple agents was explored by San Juan et al. [6], who showed that a free formation is optimal. This means the UAVs should not be restricted to traverse a pre-specified area but should roam free. Similarly, when allowing for different start/end nodes, a better route could therefore be obtained, but the costs of changing those should be integrated.

The TOP formulation is widely applied with many extensions. A time-dependent orienteering problem with time windows was explored by Verbeeck et al. [18]. In the case of the UAV path, planning this is relevant during a natural disaster or when a given weather forecast only allows for partial flight allowing for access to different areas at different times.

Another important aspect is safety, as obstacles such as geographic obstructions, housing, or uncertainty in GPS precision and speed should be considered. In the works of Lee et al. [19] an optimal path was found while considering collision while, e.g., Sung et al. [20] consider online updating. This is a field on its own as this needs a discussion of online vs. offline planning.

Poggi et al. [9] presents a multitude of TOP formulations. In one formulation, the number of variables is polynomial, while the number of constraints is exponential. The second formulation is less compact than the first and has a pseudo-polynomial number of variables and can be seen as a flow formulation. The third is the column generation formulation which is used for the Branch-Cut and Price algorithm presented. The main takeaway of this is the importance of merging the theoretical framework with the specific problem characteristics.

As the TOP model has been successful in many applications, it will be used to tackle the SAR problem in this research contribution. In particular, it fits the setup with the UAVs representing the agents and the goal of maximising scores, which reflect how likely it is to find a target at a location. Other aspects such as the time budget and maximum travel time can also be embedded, making it easy to represent the problem as a TOP and thereby increases the likelihood of generating solutions that are near-optimal when translated to reality.

## 3. Problem Formulation

To facilitate a successful solution approach, the corresponding mathematical model for the problem is first formulated. In this context, it must be noted that the formulation differs from the one used in, e.g., [9], as the constraints on arc usage and subtours have been altered. This increases the realism of the proposed model and fitness to SAR missions.

The SAR problem is formulated mathematically with the goal being to locate as many targets as possible, while only having knowledge of the scores of each cell. Note, the score indicates the likelihood of acquiring a target. This means the problem is indirectly formulated such that the likelihood of locating as many targets as possible is maximised. As nodes are either visited or not, the problem can be formulated through Mixed Integer Programming (MIP).

To limit the scope of this study, it is assumed that: (1) a list of potential target locations is available and can be used to generate a score map; (2) the score map is static, i.e., the target locations and their scores do not change over time; (3) the targets are observed with certainty; (4) the score map is 2-dimensional, i.e., terrain distinction and obstacles are not considered; and (5) UAVs with intersecting paths will not collide. In practice, it is assumed that UAVs navigate between search areas at different altitudes.

The problem is formulated as a TOP in this research. This choice is due to the simple representation of fleet and constraints on the manoeuvring. In short, the TOP introduces a network and a fleet of agents that can travel to a subset of nodes with an associated score. The main objective is to accumulate the highest possible total score. The formulation is similar to the one found in Poggi et al. [9]. Except, in this formulation the constraint on arc usage is removed due to it being redundant when constraint (2) is introduced. Additionally, the *DFJ* approach for subtour constraints is exchanged with the subtour constraint proposed by Vansteenwegen et al. [21]. The new subtour constraint is based on the *MTZ* method [22], where a constraint is added for each pair of nodes. Thereby, adding a constraint for each subtour, as the *DFJ* approach would, can be avoided. This ultimately avoids an exponential increase in the number of constraints [23,24] but results in a polynomial increase instead.

The following notation is used in the formulation:

- *N* is the set of nodes *v* in the search area.
- $y_{v}$ is a binary variable indicating whether node *v* has been visited.
- $S_{v}$ indicates the relative value of the node *v*.
- An arc is denoted as *a*, and the set of all arcs in the network is denoted as *A*.
- The arc $a_{\left(v,w\right)}$ is the arc from node *v* to node *w*.
- $l_{a}$ is the length of arc *a*.
- $\delta^{-}\left(v\right)$ denotes the arcs that end in *v*, and $\delta^{+}\left(v\right)$ are the arcs that start in *v*. They are commonly referred to as in-degree and out-degree.
- The agents start and end at the base node $v_{0}$.
- *m* is the number of agents.
- $x_{a}^{k}$ is a binary variable that indicates whether arc *a* is traversed by vehicle *k*.
- Agent *k* can travel a maximum distance of $L^{k}$.
- $u_{v}^{k}$ is a continuous variable used for the subtour constraints.

Note that the base, $v_{0}$, is located at the point (0,0) of the map. Using this notation, the TOP for SAR route planning can be represented as the following MIP.

(1) $$\begin{array}{cccccc} \max_{x,y,u}\; & \sum_{v\in N}S_{v}\cdot y_{v} & & \; & & \end{array}$$

(2) $$\begin{array}{cccccc} s.t.\;\; & \sum_{k=1}^{m}\sum_{a\in \delta^{-}\left(v\right)}x_{a}^{k}-y_{v} & & =0\; & & \forall v\in N \end{array}$$

(3) $$\begin{array}{ccccc} & \sum_{a\in A}l_{a}x_{a}^{k} & \leq L^{k}\; & & k=1,\cdots ,m \end{array}$$

(4) $$\begin{array}{cccccc} & \sum_{a\in \delta^{+}\left(v_{0}\right)}x_{a}^{k} & & =1\; & & k=1,\ldots ,m \end{array}$$

(5) $$\begin{array}{cccccc} & \sum_{a\in \delta^{-}\left(v_{0}\right)}x_{a}^{k} & & =1\; & & k=1,\ldots ,m \end{array}$$

(6) $$\begin{array}{cccccc} & \sum_{a\in \delta^{-}\left(v\right)}x_{a}^{k}-\sum_{a\in \delta^{+}\left(v\right)}x_{a}^{k} & & =0\; & & \forall a\in A\;\forall k=1,\ldots ,m \end{array}$$

(7) $$\begin{array}{cccc} \; & 2\leq u_{v}^{k}\leq \left|N\right|\; & & \forall v\in N\;\forall k=1,\cdots ,m \end{array}$$

(8) $$\begin{array}{cccc} & u_{v}^{k}-u_{w}^{k}+1\leq \left(|N|-1\right)\left(1-x_{a_{\left(v,w\right)}}^{k}\right)\;\; & & \forall a_{\left(v,w\right)}\in A\;\forall k=1,\cdots ,m \end{array}$$

(9) $$\begin{array}{cccc} & y_{v}\in \left\{0,1\right\}\; & & \forall v\in N \end{array}$$

(10) $$\begin{array}{cccc} & x_{a}^{k}\in \left\{0,1\right\}\; & & \forall a\in A\;\forall k=1,\ldots ,m \end{array}$$

The objective function (1) is the cumulative score to maximise and the criteria by which the optimal solution is evaluated. Constraint (2) ensures that each node is visited at most once by at most one vehicle. Constraint (3) is the travel budget, which represents the range of the UAVs. Constraints (4) and (5) force the vehicles to start and end at the base. Constraint (6) is the flow conservation constraint, which ensures that all nodes are left the same number of times that they are entered. Constraints (7) and (8) are the subtour constraints which ensure that the entire path for each vehicle is fully connected. The intuition is that $u_{v}^{k}$ must have a lower value than $u_{w}^{k}$ if node *v* is visited before node *w* by vehicle *k*. If a node $v^{\prime }$ is not visited by vehicle *k*, then the variable $u_{v^{\prime }}^{k}$ is free to be any value between 2 and |N|. Finally, constraints (9) and (10) are the binary constraints, ensuring that nodes and arcs are either visited or not.

The proposed mathematical formulation only has linear constraints, but the number of constraints and the size of the search space make the optimal solution very difficult to obtain when problem scenarios are not small. In other words, this means that it is not possible to guarantee finding an optimal solution in polynomial time, unless P=NP. More precisely, The TOP formulation is in NP as solutions can be evaluated in polynomial time. Furthermore, the TOP is shown to be NP-hard as it is a generalisation of the Orienteering Problem (OP) [9,25]. Therefore, it is NP-complete. Due to the computation complexity of the TOP, exact solutions are only explored for small instances, and heuristic solutions are considered for large-scale instances.

## 4. The Proposed Approach

Planning time is crucial due the urgency of SAR mission planning scenarios. As a result of this and of the scale of the problems, exact solution approaches are deemed unfit for any realistic problem to avoid that rescue turns into recovery. However, an exact solution approach can still operate as a benchmark in the evaluation of other solution methods on small problem instances.

### 4.1. Exact Solution Approach

There are numerous open source and commercial solvers able to find an exact solution to MIP problems. In this study, the IBM ILOG CPLEX optimiser which uses a Branch and Cut method is utilised.

**Figure 2:**
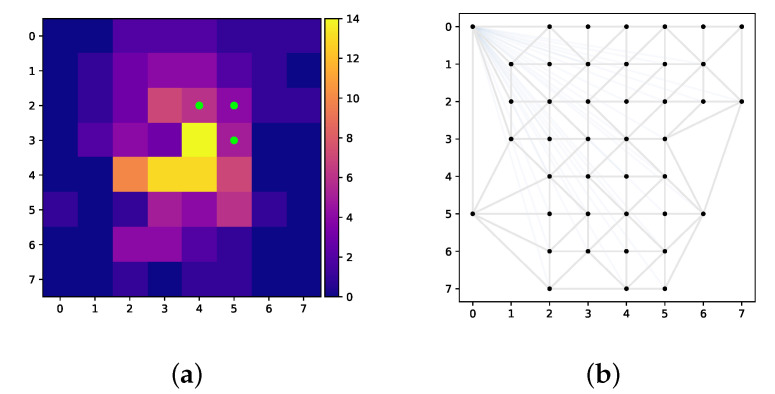
Illustrations of a small map instance. Each tile of the map is 0.5 × 0.5 km. (**a**) Score map. (**b**) Graph representation.

Herein, an exact solution to the map instance in Figure 2 is presented. A neighbourhood level of 0 is considered for a scenario with one vehicle and the ranges 10 km, 15 km and 20 km.

**Figure 3:**
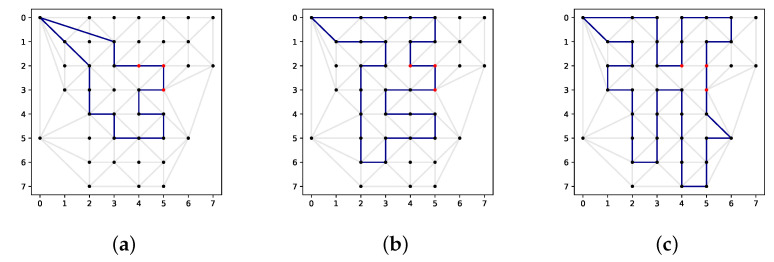
Exact solutions of the small instance. (**a**) Length constraint of 10 km. (**b**) Length constraint of 15 km. (**c**) Length constraint of 20 km.

The solution paths are shown in Figure 3, and the numerical results are reported in Table 1. In the figure, it can be clearly seen that all paths are successful in locating the 3 targets. The values of the objective function vary from 106 to 145 amounting from 70.7% to 96.7% of the possible score. Computation times of 1772 and 1635 s for the ranges of 10 km and 15 km are observed, respectively, whereas for the range of 20 km, the solution took 5801 s to find. This is a major drawback of this exact approach as it makes it virtually infeasible for more realistic problem instances (higher number of UAVS, higher granularity, etc.).

### 4.2. Heuristics and Metaheuristics

As the exact solution approach was found to be computationally infeasible even for small instances, it is necessary to consider faster solutions even if they are not optimal. In particular, heuristics and metaheuristics are considered in this research. However, the efficiency of these methods is not guarantied, as each problem instance can be very different, and the balance between avoiding local optima traps while still converging towards an optima fast gets increasingly difficult. Ultimately, one wants a method that exploits the problem structure [26]. The No Free Lunch theorem clearly states that there is not one universal method that will outperform others for all optimisation problems [27]. Metaheuristics are not problem specific as they supply a generic framework. Therefore, domain knowledge can be introduced to improve the performance of these methods, mainly in terms of the runtime and memory usage, for instance, to carefully choose the problem representation, the solution format, and adequate data structures and to embed as much domain knowledge as possible. Herein, heuristic and metaheuristic methods are proposed as solution approaches to tackle the SAR problem formulated in Section 3, in addition to a constructive algorithm which is used in the proposed metaheuristic framework.

#### 4.2.1. A Greedy Algorithm

One of the fastest and common solution approaches are greedy algorithms. These algorithms work in a constructive manner to construct a solution from scratch by looking one step ahead and choosing the immediate option with best local improvement, while still returning a feasible solution. These are sometimes referred to as a myopic heuristics, due to them being “short-sighted” in their search procedure [11]. Greedy algorithms are useful for initialisation or as part of an iterative heuristic such as the Greedy Randomised Adaptive Search Procedure which will be introduced in the Section.

In the context of SAR, it computes the paths sequentially for each UAV. Although the goal is to maximise the cumulative score, In Equation (1), the greedy heuristic utilises the Score/Distance Ratio (SDR) instead of the absolute score to evaluate the immediate improvement of a move. This approach is often used in Knapsack Problems [28] and further justified by the reasonable assumption that a good solutions has to (almost) deplete the travel budget constraint (3). Furthermore, if a solution has the same cumulative score but is shorter, then there is a high likelihood that one easily could be improved to a superior solution. Note, utilising the SDR in the evaluation therefore reinforces the search characteristic of adding multiple nearby nodes rather than one high-scoring node further away.

**Figure 4:**
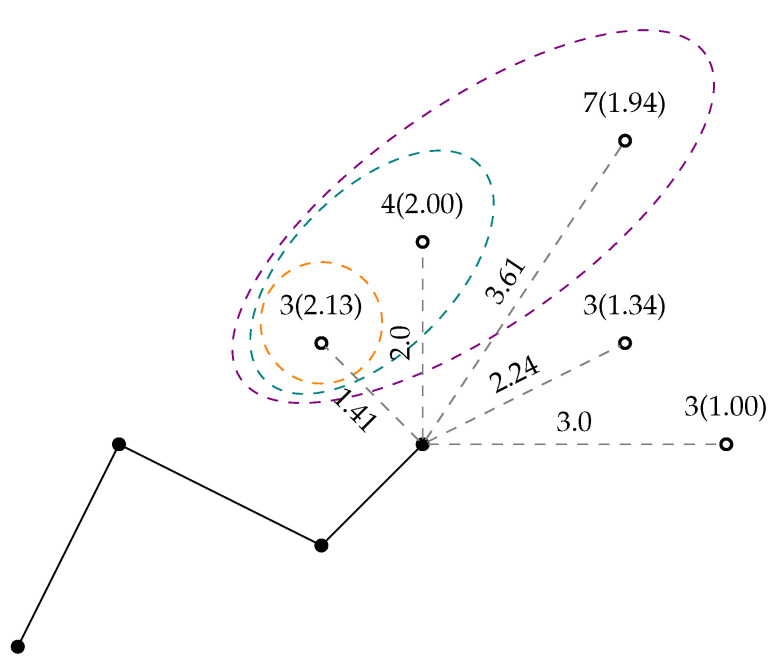
An example of a single decision cycle in the greedy heuristic. Here, the algorithm has to decide between the nodes connected with grey dashed lines. The node scores are written above with SDR in parentheses, and the arc lengths are shown next to the arcs. The coloured ellipses showcase the RCL list for 100%, 80% and 60%, respectively.

In the implementation of the greedy algorithm in this research, the Restricted Candidate List (RCL) is added, and an initial steering of solutions with clustering and a modification of the network are introduced in the event of deadlock. The indirect constraints, such as the travel distance in line 13, ensure the feasibility of the solution. The RCL parameter adds randomness to the construction phase which aids in avoiding the local optima traps. An illustration of the decision making is seen in Figure 4. In a standard greedy algorithm implementation, the RCL parameter is nullified by setting it to 1, leading to the best move always being chosen.

For initialisation purposes, the centroid parameter is introduced. If it is set to *FALSE*, the algorithm chooses the node with the highest SDR. If it is set to *TRUE*, a pre-processing step is performed on the map. Here, the map is partitioned using k-means clustering [29], where the number of available vehicles determines the number of clusters. The algorithm then chooses the nearest node to each of the geometric centroids of each cluster as initial nodes. This enforces each UAV to fly to a centroid from the beginning. Hereafter the algorithm is executed as usual. The utilisation of K-means is intended to explore the map more, as the myopic nature of the heuristic keeps it around the base. Additionally, the Dijkstra algorithm is used for finding the shortest path between nodes as it generates optimal paths in a fast manner.

When constructing the path, a deadlock could appear. Meaning it is not possible to travel anywhere from the previously chosen node, as all neighbouring nodes already have been visited. This limitation stems from constraint (2). Instead of backtracking the solution to solve the problem, a deeper level of the Delaunay triangulation is introduced. Here, the nearest unvisited node is found in the deeper levels, node *w*, the two arcs $a_{\left(v,w\right)}$ and $a_{\left(w,v\right)}$ are then added to the network. This can potentially happen a couple of times before the deadlock is solved, allowing the search procedure to continue. Note that the detailed pseudocode of the proposed greedy algorithm is given in Appendix B.

#### 4.2.2. Hill Climbing

Given a feasible solution, generated randomly or using a greedy algorithm, it is usually possible to further improve it using incremental changes through local search algorithms such as Hill Climbing (HC). These algorithms iteratively search the nearby neighbourhood for improvements. Although Hill Climbing can significantly improve solutions, it does not guarantee optimality nor high quality solutions.

The algorithm comprises two different operators: Add and Remove. The implementation is sequentially executed for each UAV. The final step of Hill Climbing is to look for nodes that improve the incumbent solution with the highest SDR. Note that the greedy algorithm has likely used most of the travel budget, and only adding nodes would therefore not be very efficient. Therefore, it is also investigated whether it is possible to remove inefficient nodes. The algorithm looks at the solution and selection of node variables, $y^{\prime }$, as a long list of binary bits. Therefore, it is possible to check for improvements in the paths total SDR by flipping one bit at a time. In the same manner as the greedy algorithm, nodes with a low SDR are removed and nodes with a high SDR are added in turn.

In practice, this procedure is repeated until the path’s SDR cannot be increased further. As a consequence, an effective route is obtained and the algorithm stops by adding the best remaining nodes until the travel budget is utilised, as the final objective is only the cumulative score.

To reduce computational complexity of Hill Climbing rather than recalculate the scores of a complete path, the impact an operation has on the incumbent solution (e.g., $\Delta_{score}$) is calculated.

The proposed Hill Climbing implementation is shown in Appendix B in addition to the the operators used for enabling or disabling nodes.

#### 4.2.3. GRASP

A natural extension of the greedy and hill climbing algorithms is the Greedy Randomised Adaptive Search Procedure (GRASP) first conceptualised by Feo and Resende [30] in 1989. GRASP is based on a modification of another algorithm by Chvatal [31]. The authors tested the algorithm on set covering problems and succeeded in generating good quality solutions. The same authors introduced a metaheuristic version of the GRASP framework [26] and detailed its usage in combinatorial optimisation problems. Although the algorithm is quite simple, it has shown strong results when compared to state-of-the-art metaheuristics such as Simulated Annealing, Tabu Search and Genetic Algorithms [26].

**Figure 5:**
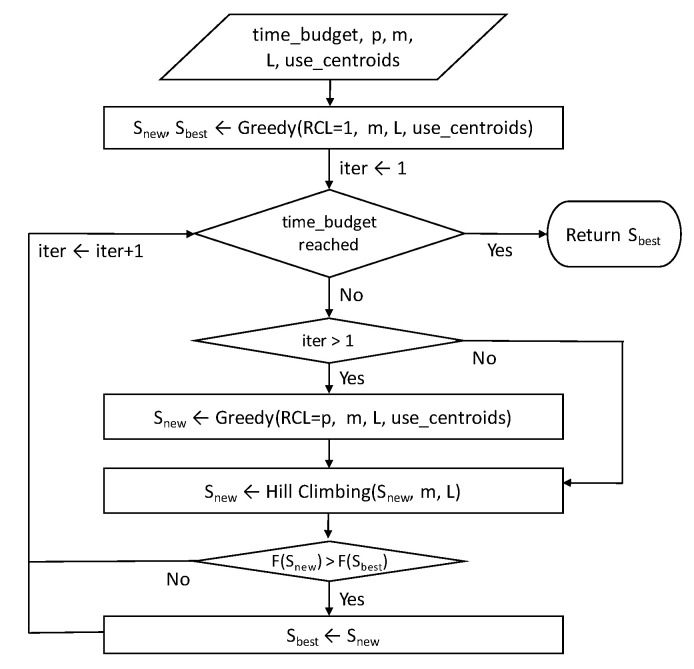
GRASP flowchart. The function F(.) represents the accumulative score.

The proposed GRASP algorithm is summarised as a flowchart in Figure 5, and its pseudocode is shown in Appendix B. The solutions are iteratively generated and improved until the time budget (time_budget) expires. Each iteration comprises a solution construction phase with a fixed RCL and a local search phase.

The RCL holds the best nodes to add at a given iteration. Here, a percentage-based approach is utilised, where, at each iteration, a number is randomly chosen among a fraction, 1−p, of the best candidate nodes. This means that the p·100% worst candidate nodes are removed from the candidate list to choose randomly from. Note the parameter p∈[0,1] is supplied by the operators. If p=1 then only the point(s) with the highest SDR are considered, and the method is therefore reduced to the standard greedy heuristic. Similarly, p=0 would be a completely random choice. When p∈(0,1), the construction is called *semi-greedy* according to Hart and Shogan [32]. There also exist a cardinality-based approach, but the number of feasible arcs connected to a node can vary greatly, which makes it prohibitively restrictive to fix a certain number of nodes to visit.

In the first iteration of the algorithm, the path construction is standard greedy (meaning RCL = 1). In extension, the first iteration serves as the lower bound, and the subsequent iterations allow to explore the solution space further by different RCL values. As all iterations are independent, the algorithm is able to compute in parallel. In that case, each core would retrieve a relatively best solution which before execution only would have to be evaluated against each other. Opposed to a traditional greedy approach that is followed by a local search, the parameters time_budget and RCL of GRASP allow for the inclusion of a global search.

## 5. Experimental Results and Discussion

### 5.1. Experimental Setting

Two different types of UAVs, corresponding to real world models, are considered throughout all experiments. The specifications of these UAVs are summarised in Table 2.

All the experiments conducted in this research are performed on an AMD Ryzen 7 4800U 8-core CPU with 16 gigabytes of RAM. Parallel processing is used by running 8 instances of the GRASP algorithm simultaneously, thus utilising all 8 cores of the CPU.

Furthermore, due to the time-sensitive nature of SAR missions, it is critical that the mission planner should be able to impose a maximum time budget on the algorithm that generates the solution. Herein, the maximum time-budget is fixed at 5 min for all instances as it is a reasonable time frame to wait while being enough for good solutions to be generated in many set-ups.

All SAR instances are generated based on a map containing a score for each location. For a detailed explanation of the procedure used for generating the instances and the graph-based representation of the problem, the reader is referred to Appendix A.

### 5.2. Parameter Tuning

**Figure 6:**
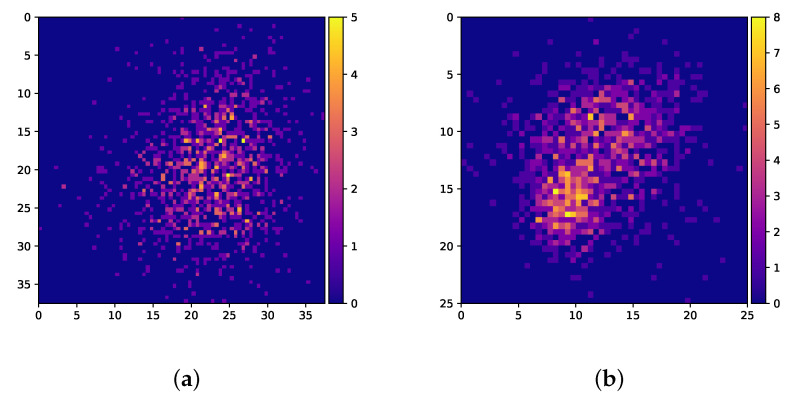
The two map instances used for parameter tuning. (**a**) Score map of the instance 11b335. (**b**) Score map of the instance 27bb50.

Parameters are determined by testing different parameter value combinations on two different problem instances. Potential parameter values are shown in Table 3, and the 2 instances maps considered are illustrated in Figure 6. Thus, 30 different combinations of parameters are tested on 2 different problem instances for a total of 60 combinations. The problem features of the two instances for which parameter tuning is performed are summarised in Table 4.

**Figure 7:**
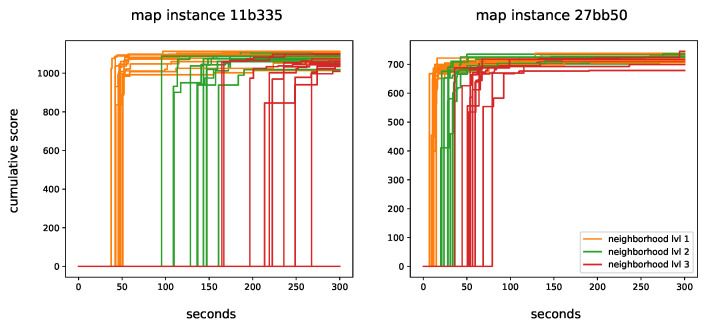
Convergence plot of all considered solutions on the two instances.

Figure 7 illustrates the convergence plots for all combinations of parameter values. The difference in the computational complexity can be clearly seen when the depth of Delaunay triangulation changes. This shows the importance of network design; if all nodes are connected, the instances would not be solvable given a reasonable time budget. Some of these combinations are not even able to find a solution for the first instance within 5 min with nghbr_lvl=3.

As the results are close, it is difficult to distinguish best performing parameters from the plots. Therefore, in Table 5, the parameters which are ranked in the top 10 are summarised. It can be clearly seen that RCL is high in all cases. This means that inducing randomness is beneficial to some extent, but the path construction should still have some guidance. In addition, all levels of neighbourhoods are covered, and both clustering and no clustering solutions showed good results. The two instance maps are quite centred in a single cluster. It is expected that clustering will perform better when the maps have irregular shapes. As it also performs well on regular maps, clustering is always utilised.

The figure also shows that the difference in the cumulative score is rather small between the methods, especially on the smaller instance 27bb50. For nghbr_lvl=1, more iterations are be completed, which expands the search space. For nghbr_lvl=2, fewer iterations are completed as expected. However, it seems that the larger solution space is easier to exploit and good solutions are identified faster. Similarly, nghbr_lvl=3 leads to less iterations of GRASP, which causes the algorithm to be slower to converge or to not converge at all in some instances.

**Figure 8:**
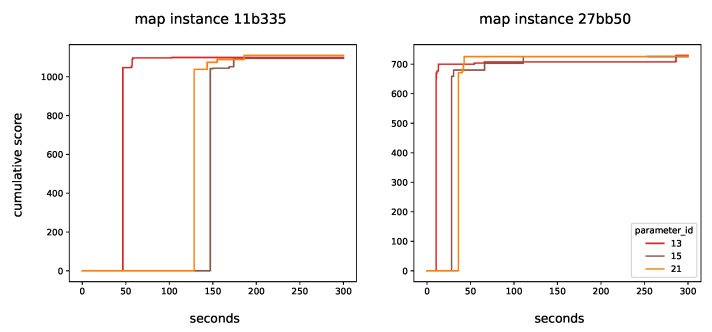
Convergence plot for the three remaining solutions.

Figure 8 illustrates the convergence plot of the three remaining methods, namely, 13, 15 and 21. There is not a benefit that strictly favours any of these three methods. It can be seen that the advantages of a neighbourhood level of 2 outweighs the drawbacks. Generating good solutions may take a bit more time. Note that as the number of UAVs increases, it is undesirable to allow their paths to cross, which might happen if nodes are not well connected. Parameter 21 is chosen over Parameter 15 as it dominates it in in both instances in terms of the cumulative score. Therefore, the following parameters are selected as the final outcome of the tuning process.

- RCL: 0.8;
- nghbr_lvl: 2;
- use_centroids: TRUE.

### 5.3. Numerical Results

To compare the three proposed methods, experiments are conducted where each algorithm is run on 9 different problem instances, varying in size. The size of the instances varies from 10 to 50 in increments of 5 km. To increase the difficulty of the instances, each node in a map is made to represent a 0.5×0.5 km area. For instance, given a 25×25 km area, a map with 50×50 tiles is generated. The size of the instance is used to decide the number and type of UAVs to consider. Regarding the type of the UAVs, the following scheme is considered:

- Short range UAVs are used for the three smallest instances.
- An even mix of short and long range UAVs is considered for the next three instances.
- Long range UAVs are used for the three largest instances.

**Figure 9:**
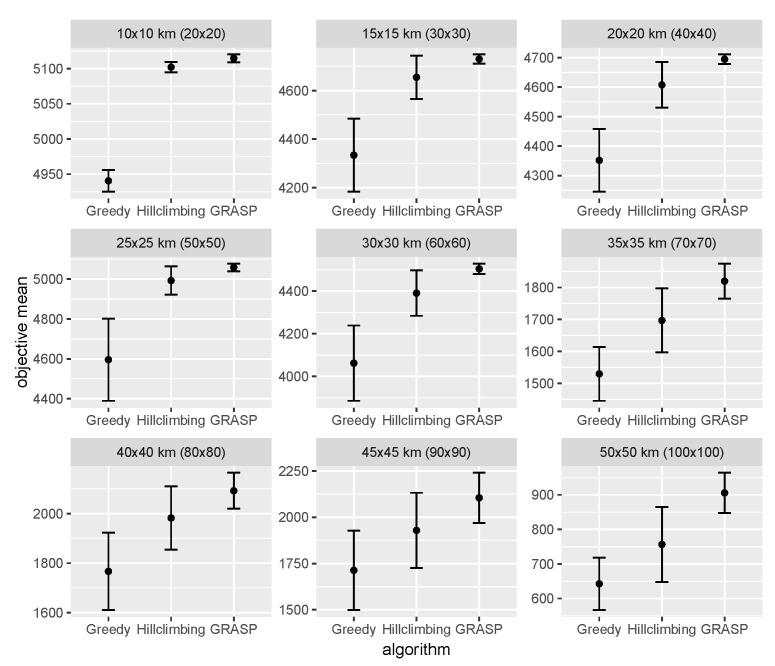
Mean objective values with standard deviation from 30 runs across the 9 instances and 3 algorithms.

The results from these experiments are summarised in Figure 9. For more detailed numerical results, including the algorithms’ runtimes, the reader is referred to Appendix D.

Results for the Branch and Cut solution method are not included as they are computationally infeasible for maps greater than 8×8. Therefore, this exact method is not useful for practical applications or comparison purposes.

The results show that GRASP outperforms both the Greedy and Hill Climbing algorithms for all instances. Compared to the Greedy approach, GRASP offers an average objective increase of 15.9%. When compared to the Hill Climbing approach, the increase in the objective value is 5.5%. Look at the standard deviation of the objective values, GRASP offers a 62.2% reduction compared to the Greedy approach and a 55.5% reduction compared to the Hill Climbing approach. This means that GRASP is statistically more consistent in finding solutions with similar objective values.

### 5.4. Discussion

The decisions made regarding the path planning of SAR missions can be split into long-term and short-term decisions. On the one hand, long-term decision are made before the incident triggering the mission takes place based on in-depth investigations. These decisions can be (1) *Strategic*, such as the establishment of base locations, determination of mission types and study of the history of incidents in a given region, or (2) *Tactical*, such as deciding the quantity, type, and properties of UAVs to purchase. On the other hand, short term decisions are made after an incident happens and are usually operational in nature. Such decisions can include UAV-task assignments and number of UAVs to allocate for the mission and path planning. Based on that, the contribution in this research can be positioned in the latter.

Manoeuvreability of the UAVs is an aspect that has not been covered in this paper. The heterogeneous UAVs may have different manoeuvreabilities, which depends on their individual construction. Those with many rotors can turn sharp corners, while plane-shaped constructions will turn in longer arcs. In order to overcome this limitation, one could construct a function that evaluates the angle acuteness of all arc pairs and add a constraint for the angle in the problem formulation. Otherwise, a penalty of the angle could be used in the objective function.

Alternatively, a preprocessing step can be implemented to prune the generated map of infeasible arc pairs and make these unavailable before solving the problem. If the area is frequently searched and the structure of the area is already known, this can even be executed before the search task occur, moving the decision from operational to tactical. These approaches will keep the problem linear even if the constructed function is not.

**Figure 10:**
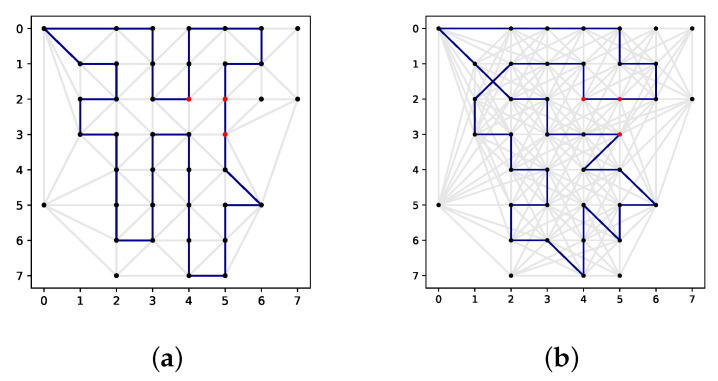
Exact and heuristic solutions to map instance e09dbf. (**a**) Optimal solution. (**b**) GRASP solution.

Figure 10 illustrates an optimal solution and a solution generated using GRASP on the same 8×8 map from Section 4.1. The optimal solution has a cumulative score of 145, and the GRASP solution has a cumulative score of 144, which is less than 1% in difference. Note that the optimal solution uses the lowest neighbourhood level of 0—due to the high computational complexity of Branch and Cut—where GRASP uses a neighbourhood level of 2 based on the tuning outcome, which does change the solution space. Since the optimal solution is more likely to exploit scores of nearby nodes, it is not expected that a larger solution space for the map would yield a different a higher cumulative score by using Branch and Cut. However, this can be useful for metaheuristics as it relieves the myopic tendencies to improve the local searches. Clearly, the solution space is much smaller in this example compared to other maps studied in this research. Therefore, the optimality gap would not scale well. Nevertheless, it is interesting to see that on this small network, GRASP produced a near-optimal solution in a tight time-budget of 5 min, where CPLEX needed 1.5 h.

GRASP clearly scales better compared to exact approaches due to its polynomial computational complexity. Nevertheless, this comes with the challenges of finding a suitable algorithm configuration. The parameters of GRASP are tuned in a way to increase the algorithm’s performance in terms of the objective value. However, SAR problems can have significantly different features given the scenario at hand. Thus, fixing the parameters leads to the risk of using a sub-optimal algorithm configurations. For instance, a neighbourhood level of 3 does not allow the algorithm to convergence for the large map as shown in Figure 7. However, convergence can be reached for smaller maps without the need for increasing the time budget. Thus, considering a deeper neighbourhood level can increase the potential objective values. A good alternative would be to allow the parameters to be expressed in terms of the problem features, which would require testing for all parameter combinations on a larger set of instances, which can be seen as a second layer optimisation problem [33]. Feature-dependent parameter tuning is a potential solution to this issue, and multiple research directions are being explored to achieve this goal using regression methods [34], machine learning [35], and genetic programming [36].

In this research, the search radius in which targets will always be spotted is assumed to be constant for all UAVs, in which the targets will always be spotted. It is possible to improve optical sensors, for instance through object recognition techniques, to extend the search radius. More importantly, there might be a trade-off between the search radius and the probability of finding a target in a given area.

## 6. Conclusions and Future Directions

In this research contribution, the problem of UAV path planning for Search and Rescue was presented and formulated as a MIP. Algorithms were proposed to automatically construct UAV paths that are optimised to locate as many targets as possible. Based the formulation, the problem was found to be NP-hard. Therefore, heuristics were a natural solution for most map sizes and time budgets in the SAR missions tackled in this work. Heuristic methods were presented and analysed. The choice of GRASP as a solution framework is justified through experimentation which showed that the algorithm offers a good trade-off between low computation time and good solution quality. Furthermore, the comparison with greedy and local search algorithms shows that GRASP generated better solutions for all instances and was more consistent in terms of the objective value.

Furthermore, the parameters of GRASP were further investigated. Specifically, the algorithm was tuned based on the combinations of multiple parameter values. Ideally, the parameter tuning is done on many instances or using machine learning methods to generate instance-specific parameters. However, such analyses are not the main focus of this paper, and the optimised parameters are found to be intuitive and efficient. The results show that a moderate amount of randomness (induced by RCL=0.8) and the network augmentation (nghr_lvl=2) were beneficial. Given a time budget of 5 min, these parameters seem sufficient to produce good quality solutions. Nevertheless, if the time horizon was longer, considering a higher neighbourhood level or a fully connected graph could potentially produce better solutions.

In terms of the problem modelling, the proposed model can be improved by relaxing the assumptions made in this research regarding the certainty of observing target, ignorance of weather conditions, and non-conflicting UAV routes. In particular, safety and collision avoidance can be supported through the simulation of UAVs routes given uncertainty in speed, GPS precision, wind, etc. Another interesting direction is to investigate whether it is worth using all available UAVs for a given mission, knowing that other emergencies may occur. This is another optimisation problem and may be solvable by considering the marginal effect of adding a UAV to a fleet on the cumulative score and weighing it against the probability of another accident occurring in the same time frame and how useful another UAV would be.

## Figures and Tables

**Table 1:** Time spent and other statistics from the three different exact solutions.

**Range constraint, km**	10	15	40
**Time to solve, s**	1771.8	1634.8	5801.06
**Objective, cumulative score**	106	134	145
**Share of total score**	70.7%	89.3%	96.7%

**Table 2:** Specification of the UAV models represented in the project.

Specification	UAV1	UAV2
**Name**	Sentaero BVLOS	UVH-25EL
**Max speed, km/h**	70	100
**Flight time, min**	72	90
**Range, km**	84	150

**Table 3:** GRASP parameters and their considered values.

Parameters	Values
RCL	{0.2,0.4,0.6,0.8,1}
nghbr_lvl	{1,2,3}
use_centroids	{TRUE,FALSE}

**Table 4:** The features considered for parameter tuning for the two problem instances.

Features	Instance 1	Instance 2
**Instance name**	11b335	27bb50
**Dimension of graph, tiles**	75 × 75	50 × 50
**Dimension of map, km**	37.5 × 37.5	25 × 25
**Time budget**	300	300
**Number of UAVs**	5	2
**Heterogeneous UAVs**	FALSE	FALSE
**Range of UAVs, km**	(150, 150, 150, 150, 150)	(84, 84)

**Table 5:** Top 10 ranked parameters values.

Parameter	RCL	neighbour_lvl	use_centroids
13	0.6	1	TRUE
15	0.6	2	TRUE
18	0.8	1	FALSE
20	0.8	2	FALSE
21	0.8	2	TRUE
28	1	3	FALSE

**Figure A1:**
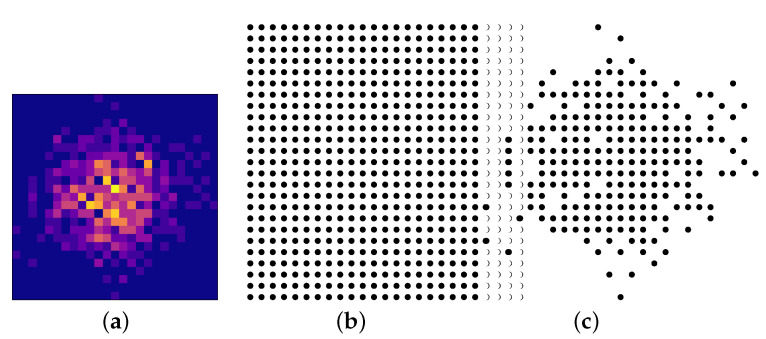
SAR score map representation with a grid of nodes. (**a**) An example of a score map with 25×25 tiles. Colours represent scores ranging from purple (low scores) to yellow (high scores). (**b**) Grid representation of the score map. (**c**) Filtered grid without nodes of score 0.

**Figure A2:**
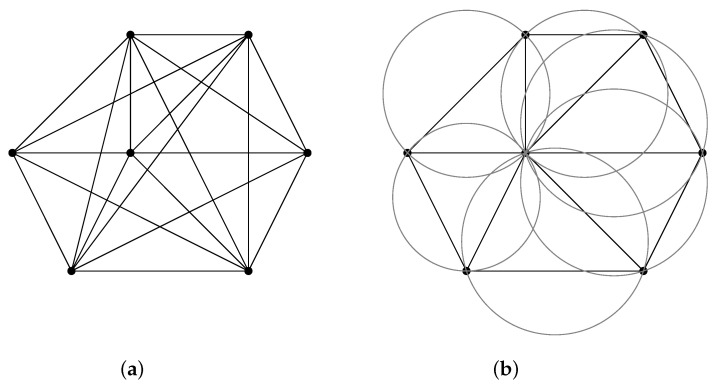
Comparison of a Delaunay triangulation and a complete graph on a small example. (**a**) Complete graph. (**b**) Delaunay triangulation with circumcircles.

**Figure A3:**
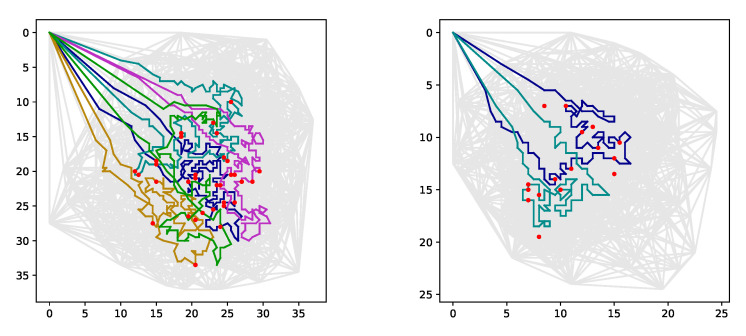
Solution of two instances of the problem using GRASP.

## Data Availability

The source codes for the proposed algorithms and problem instances are publicly available at https://github.com/Rosenkrands/sar-path-planning (accessed on 25 November 2021).

## Appendix A. Networks Representation and Map Generation

The maps considered in this study are generated as follows: a map size is first specified. Then, random values are sampled from a distribution whose parameters is a function of the dimension. For diversification, this is done for multiple centres around the map, and the scores are constructed based on a 2-dimensional histogram. Increasing the size of centres and their number will result in a more even distribution of scores across the map, making the optimal solution more difficult to identify.

For larger and therefore more realistic problems, using a grid-based representation quickly becomes computationally demanding, especially for exact solutions. For this reason, the grid-based representation is not considered here. Instead, all points with no associated score are identified and discarded. This is illustrated in Figure A1. This retains all relevant information while removing redundant complexity.

The other important aspect of the map is its connectivity which determines how the UAVs move between the nodes. Considering a fully connected network (connecting all *n* nodes with each other) results in (n−1)! possible solutions, when all possible $\frac{n(n-1)}{2}$ edges are used, which is undesirable. To overcome this issue, the Delaunay triangulation [10,37] is applied in order to reduce the graph complexity by limiting the number of edges to 6 edges on average for each node [10,37]. Specifically, the method produces triangles from nodes such that each triangle’s circumcircle does not contain other nodes. The edges of the resulting triangles compose the arcs in the network. Figure A2 is an illustration of a complete (fully connected) graph and the corresponding simplified graph using the Delaunay triangulation.

Using this setup, the obtained graph has nodes with a different number of edges, while still being a connected graph. Therefore, the network has a non-trivial topology and can be called a complex network. It can be advantageous to slightly and strategically expand the connectivity of the graph because of arc restrictions and direct travelling. Using dynamic programming, it is possible to connect a node to distant neighbours instead of connecting it only to immediate neighbours. In other words, a node can be connected to neighbouring triangles, or neighbours of neighbouring triangles, and so on. The closeness of two nodes can be measured by the number of intermediate triangles that connects them. Herein, the terminology *neighbourhood level* (or nghbr_lvl) is used as a measure of distance between two nodes. In addition, arcs are added such that it is always possible to fly directly from any node and back to the base station.

## Appendix B. Detailed Pseudocodes

This appendix contains the pseudocodes of all the proposed algorithms. Namely, the greedy Algorithm A1, the Hill Climbing Algorithm A2, the Hill Climbing operators Algorithm A3 and Algorithm A4, and the main GRASP Algorithm A5.

**Algorithm A1:** Greedy Heuristic
**Input**: *p*, Number of UAVs, Range of each UAV, use_centroids

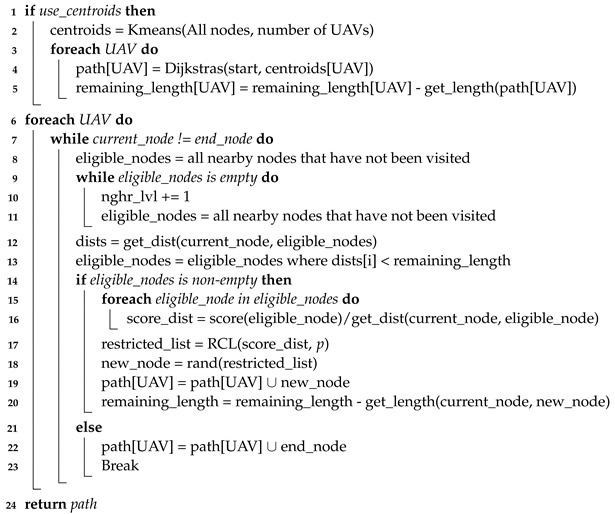

**Algorithm A2:** Hill Climbing
**Input**: sol${}_{0}$, num_uavs, ranges

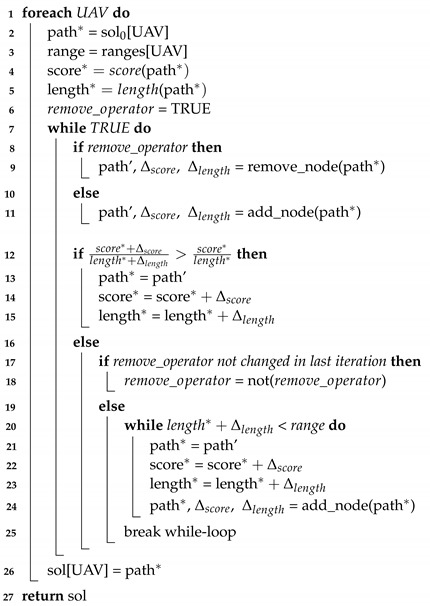

   For Algorithm A3 and Algorithm A4, when nodes are added, only the adjacent nodes of the path are considered. This is done to avoid complicating the algorithm by adding multiple nodes at a time. This also improves the computational complexity, as the number of nodes to consider is smaller.

**Algorithm A3:** Add Operator
**Input**: path${}^{*}$, remaining_length

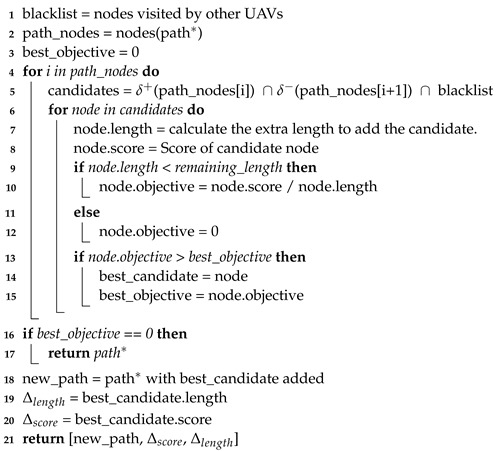

**Algorithm A4:** Remove Operator
**Input**: path${}^{*}$

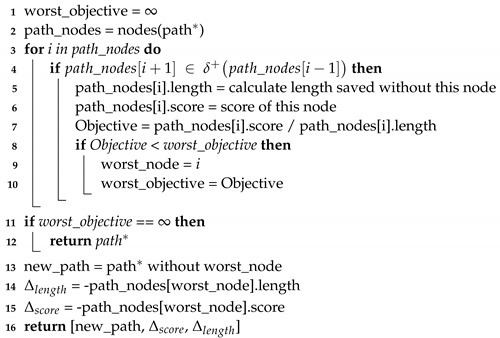

**Algorithm A5:** GRASP
**Input**: time_budget, p, num_uavs, ranges, use_centroids

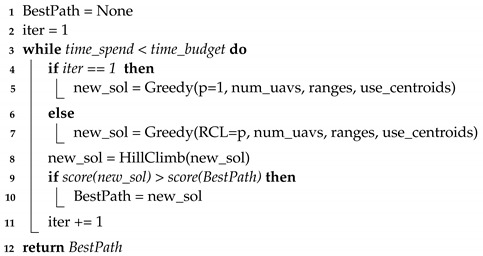

## Appendix C. Illustrations of SAR Plans Generated Using GRASP

Figure A3 shows the solutions generated using the proposed GRASP method for two SAR scenarios.

## Appendix D. Detailed Numerical Results

In this appendix, we report the numerical results in terms of the objective values and the runtimes in Table A1.

**Table A1:** Values represent the objective values and runtime respectively, and the subscripts represent the standard deviations. The sizes are expressed in km.

Objective Value					Run Time		
**Tiles**	**Size**	**GRASP**	**Greedy**	**HC**	**GRASP**	**Greedy**	**HC**
20 × 20	10 × 10	$5114.7_{5.6}$	$4940.5_{15.4}$	$5102.2_{7.4}$	$251.9_{14.2}$	$6.1_{3.0}$	$71.6_{12.9}$
30 × 30	15 × 15	$4730.3_{19.1}$	$4333.9_{150.4}$	$4655.1_{89.7}$	$272.0_{29.9}$	$6.0_{3.9}$	$80.6_{13.4}$
40 × 40	20 × 20	$4694.5_{16.5}$	$4351.7_{106.2}$	$4607.6_{77.3}$	$293.2_{0.8}$	$0.2_{0.0}$	$6.7_{0.8}$
50 × 50	25 × 25	$5058.3_{19.6}$	$4596.0_{206.2}$	$4992.6_{70.8}$	$290.8_{3.0}$	$0.3_{0.5}$	$14.3_{2.9}$
60 × 60	30 × 30	$4504.3_{24.7}$	$4062.3_{176.5}$	$4390.5_{106.4}$	$279.9_{11.0}$	$0.6_{0.4}$	$125.9_{27.2}$
70 × 70	35 × 35	$1819.8_{55.1}$	$1529.6_{84.3}$	$1696.9_{100.2}$	$261.4_{24.2}$	$0.4_{0.2}$	$105.0_{42.4}$
80 × 80	40 × 40	$2092.2_{72.8}$	$1766.9_{156.1}$	$1982.4_{128.1}$	$253.9_{17.7}$	$0.8_{0.6}$	$118.7_{32.4}$
90 × 90	45 × 45	$2105.1_{135.4}$	$1713.4_{214.5}$	$1929.1_{202.8}$	$289.4_{3.8}$	$1.2_{1.0}$	$23.3_{3.8}$
100 × 100	50 × 50	$905.7_{58.3}$	$642.5_{75.9}$	$756.5_{108.5}$	$278.0_{12.9}$	$0.8_{0.5}$	$171.8_{23.6}$
